# Diet Switching and Interspecific Competition in Sympatric Steppe Ungulates Under Seasonal Resource Variability

**DOI:** 10.1002/ece3.72971

**Published:** 2026-01-29

**Authors:** Huiqin Dong, Bin Feng, Shuai Yang, Yuna Jia, Mingsen Qin, Wenke Bai

**Affiliations:** ^1^ Key Laboratory of Southwest China Wildlife Resources Conservation China West Normal University Nanchong China; ^2^ School of Ecology and Environment Xizang University Lhasa China; ^3^ Institute of Ecology China West Normal University Nanchong China

**Keywords:** diet, DNA macrobarcoding, Qinghai‐Tibetan Plateau, sympatric coexistence, ungulates

## Abstract

Understanding the mechanisms of competition and coexistence among sympatric species is crucial for deepening our understanding of interspecific interactions and informing the conservation of rare and endangered wildlife. In this study, we utilized DNA macro‐barcoding technology to analyze the seasonal dietary habits of Kiang ( *Equus kiang* ) and Tibetan Gazelle ( *Procapra picticaudata* ) in Shiqu County, Sichuan Province, aiming to investigate their resource partitioning strategies and potential competition for limited forage resources. The results showed that Kiang mainly consumed Cyperaceae and Polygonaceae in both seasons, while Tibetan Gazelle fed on Polygonaceae and Rosaceae in the warm season and shifted to Ephedraceae in the cold season. Both species exhibited significant seasonal differences in dietary composition, with Tibetan Gazelle showing greater individual variation and seasonal shifts. In addition, their dietary niche was broader in the warm season, and overlap remained high, with indices of 0.89 and 0.87 in the warm and cold seasons, respectively. The results indicate that although Kiang and Tibetan Gazelle exhibit partial dietary overlap, they mitigate interspecific competition and achieve sympatric coexistence through differential use of dominant forage species, adjustments in dietary proportions, and individual dietary flexibility; notably, Tibetan gazelles exhibit stronger ecological adaptability. This study highlights a competition–coexistence dynamic along the trophic niche axis in typical plateau ungulates, providing insights for effective conservation strategies and biodiversity conservation in plateau regions.

## Graphical abstract

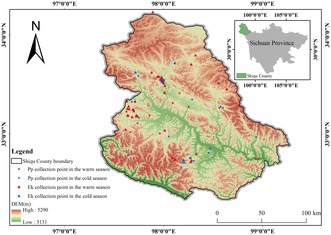

## 1 Introduction

Sympatrically distributed ungulates frequently engage in interspecific competition, particularly when their ecological niches overlap substantially (Davies 2007). The competitive exclusion principle posits that species with completely overlapping niches cannot coexist; thus, stable long‐term coexistence among sympatric species relies on ecological niche differentiation (Hardin 1960). To mitigate competition and promote coexistence, sympatric species often differentiate along temporal, spatial, and trophic niche dimensions (Carvalho and Cardoso 2020). Among these dimensions, trophic niche partitioning plays a central role in alleviating competition for limited food resources. In ecosystems where sympatric ungulates coexist, limited plant resources can intensify interspecific competition, driving the evolution of trophic niche segregation through long‐term adaptation (Zengeya et al. 2015; Prins and van Langevelde 2008). Understanding trophic niche differentiation among sympatric ungulates is essential for promoting population stability, maintaining ecosystem functionality, and guiding evidence‐based conservation strategies. However, despite growing recognition of niche partitioning, empirical studies focusing on seasonal dietary dynamics and competition mechanisms in high‐altitude ungulate communities remain limited (Li and Jiang 2007; Yin et al. 2007; Shi et al. 2016).

Wild ungulates are ecologically important taxa and serve as integral components (Reimoser and Nopp‐Mayr 2024). They play a key role in supporting populations of large and medium‐sized carnivores within trophic networks and contribute to the stability of alpine steppe and alpine meadow ecosystems (Ramirez et al. 2018; Wolf and Ripple 2016; Foster et al. 2014). Among the native ungulates of the Tibetan Plateau, Kiang ( *Equus kiang* ) and Tibetan Gazelle ( *Procapra picticaudata* ) are the most widely distributed and numerically abundant species within the alpine grassland ecosystem. Specifically, the Kiang primarily inhabits alpine steppes, wetland margins, and alpine shrublands at elevations ranging from 3200 to 5300 m (Gao and Gu 1989). By comparison, the Tibetan Gazelle occurs across a broader range of habitats, including grasslands, deserts, semi‐deserts, wetland margins, and montane shrublands, typically at elevations above 3500 m on the Qinghai‐Tibetan Plateau (Smith et al. 2010). Elucidating the patterns of resource competition and dietary differentiation between sympatric Kiang and Tibetan Gazelle is essential for maintaining population stability, safeguarding regional ecological security, and informing biodiversity conservation strategies.

Grassland ecosystems exhibit pronounced seasonal pulses in resource availability, with both plant biomass and nutritional quality shifting systematically between seasons; these dynamics profoundly shape herbivores' foraging decisions and adaptive strategies (Aikens et al. 2020; Wang et al. 2024; Mills et al. 2024). Diet switching constitutes a key behavioral response to such seasonal resource regimes, driving directional shifts in dietary composition and reshaping species' niche breadth (Harvey et al. 2024; Littleford‐Colquhoun et al. 2024; Carscadden et al. 2020). The application of scientifically robust and accurate analytical methods is essential for reliable diet analysis. Compared with traditional dietary analysis methods, molecular scatology is faster, more efficient, and provides finer taxonomic resolution (Roslin and Majaneva 2016; Pompanon et al. 2012). Currently, DNA‐based molecular identification techniques, particularly DNA metabarcoding, have become widely used in dietary studies across diverse taxa. For example, DNA metabarcoding technology has been used to examine dietary resource use in forest musk deer ( *Moschus berezovskii* ) and roe deer (*Capreolus* spp.) in the Lüliang Mountain's range (Pei et al. 2025), as well as to analyze trophic niche differentiation among three sympatric rodent species in the Meili Snow Mountain region of Yunnan (Qin et al. 2024).

Despite their ecological importance, current research on Kiang and Tibetan Gazelle has largely concentrated on single‐species aspects, such as population size, spatial distribution, and habitat preferences, while studies examining their dietary composition, seasonal variation, and interspecific interactions remain limited. This gap is particularly evident in high‐altitude ecosystems, where resource scarcity and seasonal fluctuations can intensify interspecific competition among sympatric herbivores. To address this gap, we employed DNA metabarcoding to comprehensively assess seasonal variation in food resource use and to investigate the mechanisms of interspecific trophic niche partitioning between sympatric Kiang and Tibetan Gazelle in Shiqu County, Sichuan Province. By linking dietary composition with seasonal dynamics, this study contributes to a deeper understanding of how resource‐based interactions shape coexistence among large herbivores in alpine grassland systems. The findings offer valuable theoretical insights for guiding regional conservation efforts and formulating management strategies aimed at sustaining ungulate diversity under changing environmental conditions.

## 2 Methods

### 2.1 Study Area

**FIGURE 1:**
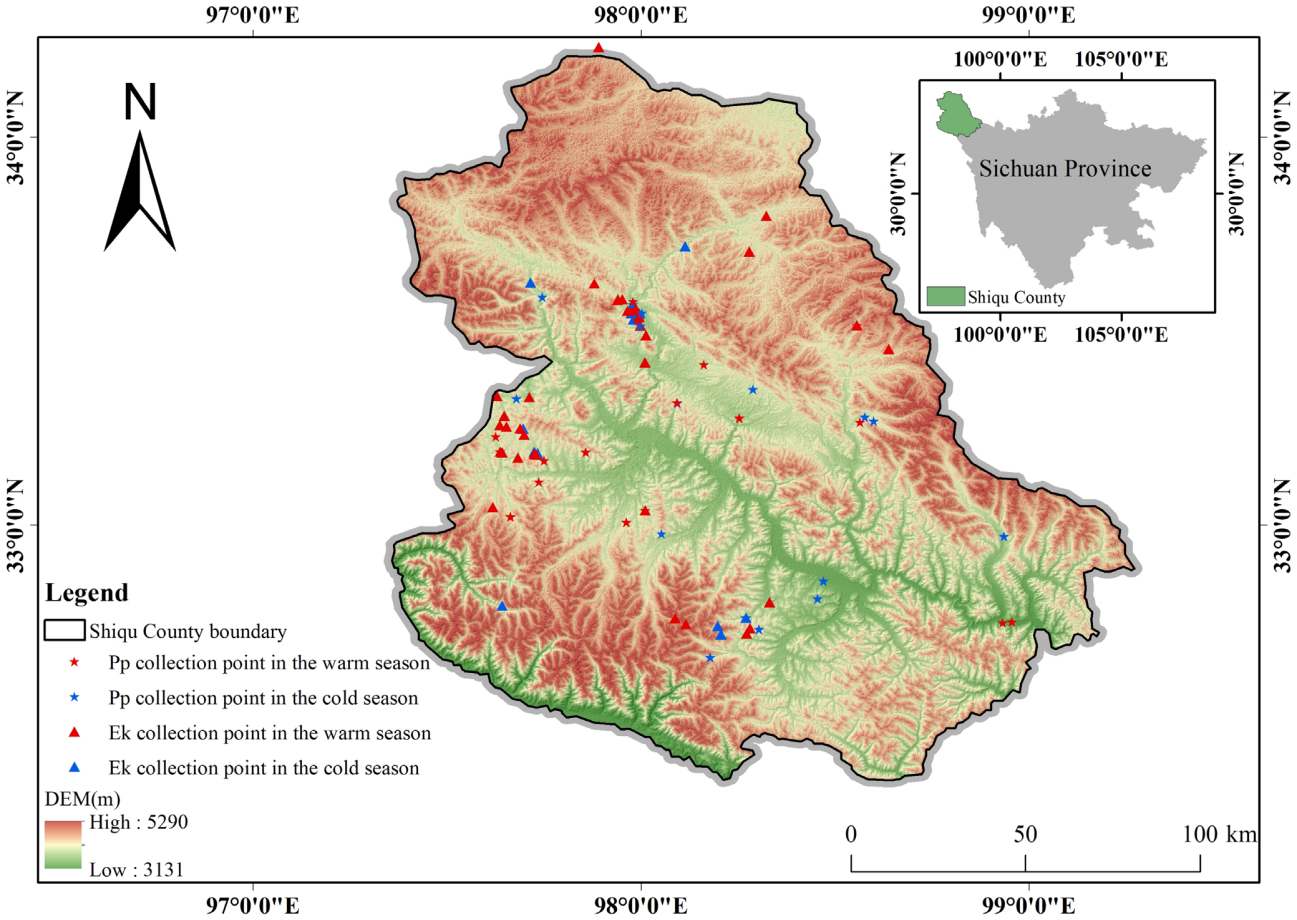
Study area and sampling site distribution.

Shiqu County is located on the northwestern edge of Garzê Tibetan Autonomous Prefecture in Sichuan Province, lying in the northern section of the southeastern margin of the Qinghai‐Tibetan Plateau (Figure 1). The county spans 25,191 km^2^, with geographic coordinates ranging from 32°19′28′′ N to 34°20′40′′ N and 97°20′00′′ E to 99°15′28′′ E, and an average elevation of 4526.9 m. The region features diverse topography and climatic conditions, supporting high biodiversity and extensive grassland resources, which cover approximately 85% of the total area. Vegetation is dominated by alpine meadows and shrubs, supplemented by alpine coniferous forests, screeside vegetation, and wetland vegetation under specific microclimatic conditions (Qiu 2006). The grasslands are rich in herbaceous species from families such as Gramineae, Cyperaceae, Ranunculaceae, Leguminosae, Compositae, Rosaceae, and Polygonaceae, with *Kobresia pygmaea* and *Kobresia setchwanensis* as representative species (Lu et al. 2005). These abundant forage resources provide a critical food base for wild ungulates. The ungulate community in Shiqu is characterized by low species richness, scattered distributions, and relatively low interspecific competition. However, significant or highly significant positive spatial associations have been observed between some species pairs, including Kiang and Tibetan Gazelle, and red deer (*Cervus elaphus sichuanicus*) and white‐lipped deer ( *Przewalskium albirostris* ) (Zhang et al. 2006). The estimated population density of Kiang is 0.41 ± 0.08 individuals/km^2^, with a population size of approximately 1395 individuals and a suitable habitat area of 3402.45 km^2^ (13.51% of the county area) (Yang et al. 2024). The Tibetan Gazelle has a lower average density of 0.117 individuals/km^2^, with a total estimated population of around 1901 individuals and a suitable habitat area of 2113 km^2^ (Lu et al. 2005; Yang et al. 2025). This region provides a natural context to investigate how sympatric ungulates partition resources and minimize trophic competition, particularly under seasonal environmental constraints.

### 2.2 Collection of Samples

We employed the line transect method to collect fecal samples across all known habitats of Kiang and Tibetan Gazelle in Shiqu County. When encountering a group of either species, we recorded population data and used binoculars to observe defecation behavior. Once defecation was confirmed, we approached the site, identified fresh feces from the observed individual, and collected samples using disposable gloves. Each sample was immediately placed in a sterilized 50 mL centrifuge tube and stored in a vehicle‐mounted freezer at −20°C. A total of 78 warm‐season fecal samples from Kiang and 36 from Tibetan Gazelle were collected during July–August 2023, while 38 cold‐season samples from Kiang and 44 from Tibetan Gazelle were obtained during March–May 2024.

### 2.3 Sequencing Data Processing

Total genomic DNA was extracted from fecal samples of Kiang and Tibetan Gazelle, and the chloroplast rbcL gene was amplified using primers Z1aF (ATGTCACCACCAACAGAGACTAAAGC) and hp2R (CGTCCTTTGTAACGATCAAG) (Hofreiter et al. 2000). Amplicons were then subjected to paired‐end sequencing. Raw sequencing data were first trimmed to remove primers using Cutadapt v2.3 (Martin 2011). Paired‐end reads were merged and filtered with VSEARCH v2.13.4 (Rognes et al. 2016). The filtered reads were then imported into QIIME 2 v2023.5 (Bolyen et al. 2019) for downstream analysis. Amplicon sequence variants (ASVs) were inferred using DADA2 (Callahan et al. 2016), which also performed quality filtering and chimera removal. ASVs shorter than 180 bp were excluded. To further enhance data reliability, additional chimera filtering was conducted using the uchime‐denovo and uchime‐ref methods implemented in VSEARCH, with the reference database provided by Bell et al. (2017). High‐quality ASVs were clustered into operational taxonomic units (OTUs) at a 97% similarity threshold, and OTUs with fewer than 200 reads were removed to retain only dominant dietary taxa. Representative OTU sequences were taxonomically annotated by aligning against the NCBI nucleotide database using BLAST (Zhang et al. 2022). Due to the high sequence similarity among closely related plant species, taxonomic resolution was restricted to the genus level. Only OTUs showing ≥ 97% sequence identity and known to occur in the local or surrounding regions were retained.

To minimize sampling bias due to sequencing depth, sample reads were rarefied to the lowest sequencing depth across all samples. This rarefaction process was repeated 1000 times, and the final diet community matrix was constructed using the average rarefied counts (rounded to the nearest integer).

### 2.4 Data Statistical Analysis

Main statistical analyses were conducted using R v.4.3.1 (R Core Team 2013). The dietary composition, relative abundance, and foraging frequency of Kiang and Tibetan Gazelle were analyzed across seasons at the taxonomic levels of order, family, and genus. To assess differences in diet composition between species within the same season and within species across different seasons, chi‐squared (*χ* ^2^) tests were conducted (Oli et al. 2018). These tests were applied to determine whether the observed differences in dietary profiles were statistically significant.

To assess the within‐group diversity of dietary composition (α‐diversity), four indices were calculated for Kiang and Tibetan Gazelle in both the warm and cold seasons, including Chao1, Shannon–Wiener index, Simpson's diversity index, and Pielou's evenness index. Differences in α‐diversity between the two species within the same season were tested using the Wilcoxon rank‐sum test (Zhou et al. 2022). To assess β‐diversity, representing differences in dietary composition between seasons and between species, Principal Coordinates Analysis (PCoA) was performed based on Bray–Curtis dissimilarity using the vegan R package (Oksanen et al. 2025). Differences in dietary composition between species and between seasons were statistically tested using permutational multivariate analysis of variance (PERMANOVA), implemented via the adonis function in vegan. To identify food items showing significant differences between Kiang and Tibetan Gazelle within the same season, as well as between seasons for each species, linear discriminant analysis effect size (LEfSe) was employed to detect taxa with significantly different relative abundances between groups (Segata et al. 2011). Our focus was on identifying groups with a *p*‐value lower than 0.05 and an LDA score exceeding 3.5 at various classification levels.

The standardized Levins' niche breadth index (Levins 1968; Colwell and Futuyma 1971) was used to describe the dietary niche breadth of Kiang and Tibetan Gazelle in different seasons.

The formula is as follows:

(1) $$\mathrm{BA}=\frac{\frac{1}{\sum {\left(P_{i}\right)}^{2}}-1}{n-1}$$

where BA represents the standardized niche breadth, and *P*_*i*_ represents the frequency of occurrence of plant species *i* in the diet of the Kiang and Tibetan Gazelle, and *n* is the total number of plant species consumed. The standardized dietary niche breadth (BA) ranges from 0 to 1; higher values indicate broader dietary niches (Colwell and Futuyma 1971).

The Pianka's overlap index (*O*_*jk*_) was employed to assess the magnitudes of the dietary niche overlap of Kiang and Tibetan Gazelle across different seasons (Pianka 1973).

The formula is as follows:

(2) $$O_{\mathrm{jk}}=\frac{\sum P_{\mathrm{ij}}P_{\mathrm{ik}}}{\sqrt{\sum P_{\mathrm{ij}}^{2}\sum P_{\mathrm{jk}}^{2}}}$$

where *P*_*ij*_ and *P*_*ik*_ represent the frequency of occurrence of food item *i* in the diets of species *j* and *k*, respectively. The value of *O*_*jk*_ ranges from 0 to 1; according to Krebs' criteria, an *O*_*jk*_ > 0.3 is considered to indicate meaningful overlap, while *O*_*jk*_ > 0.6 is regarded as significant overlap (Krebs 1999).

## 3 Results

### 3.1 Dietary Composition and Seasonal Variations of Kiang and Tibetan Gazelle

**FIGURE 2:**
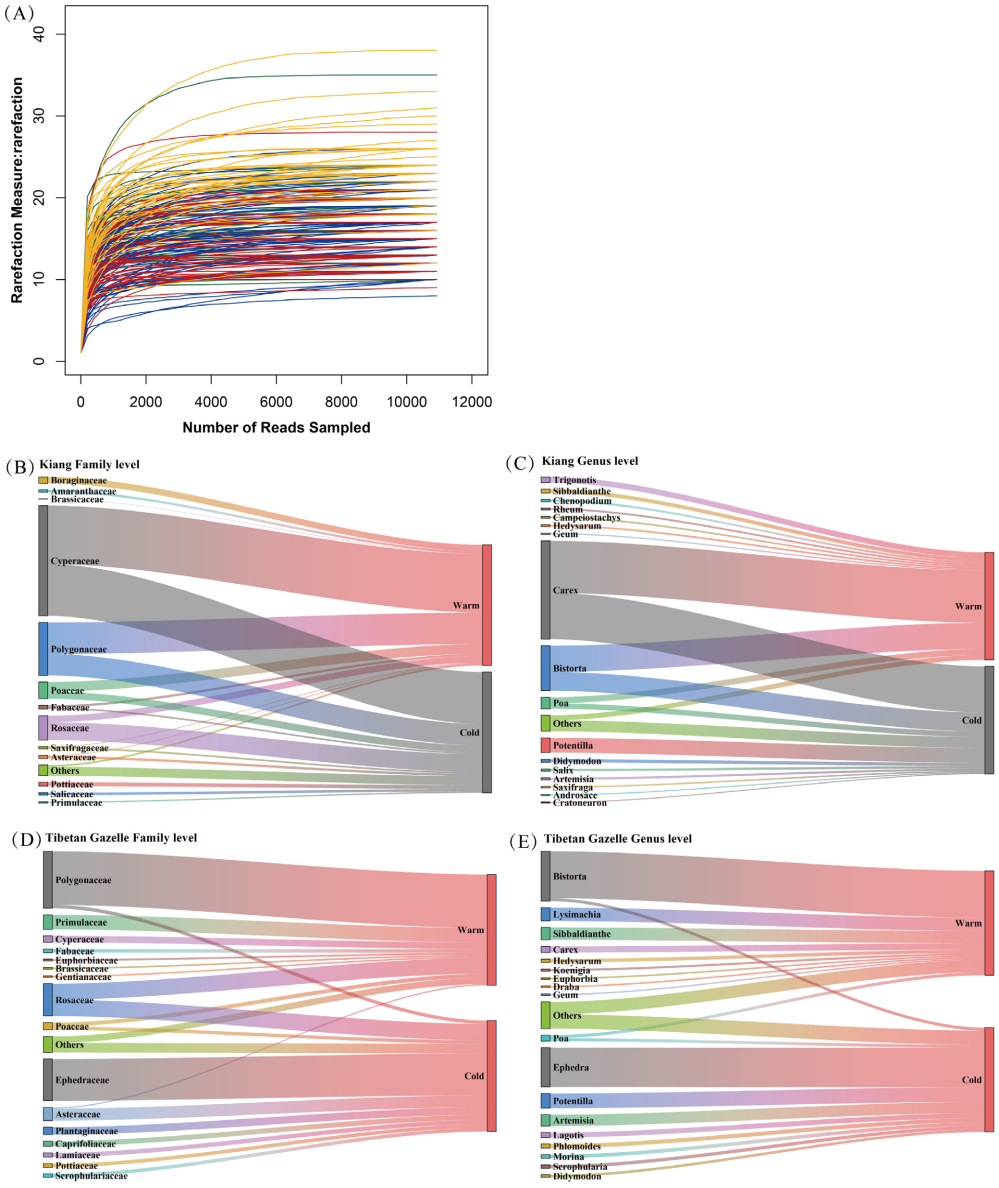
Rarefaction curves of all samples based on DNA metabarcoding data (A). Dietary composition of Kiang ( *Equus kiang* ) and Tibetan Gazelle ( *Procapra picticaudata* ) during the warm and cold seasons, analyzed at the family (B, D) and genus levels (C, E).

Based on DNA metabarcoding sequencing data, a total of 2,139,441 valid sequences were obtained from 196 samples, with an average of approximately 10,916 sequences per sample. As sequencing depth increased, the rarefaction curves gradually reached a plateau, indicating that the sequencing depth in this study was sufficient and reasonable (Figure 2A).

By referencing to the local species list, 27 orders, 36 families, and 56 genera of plants were identified in the warm‐season fecal samples of Kiang, while 25 orders, 37 families, and 63 genera of plants were identified in the cold‐season fecal samples. At the family level, the dominant plant taxa consumed by Kiang in the warm season include Cyperaceae (48.43%), Polygonaceae (26.19%), Poaceae (7.97%), Rosaceae (5.37%), and Boraginaceae (5.37%); while in the cold season, the main foraged plants include those from Cyperaceae (42.97%), Polygonaceae (17.80%), Rosaceae (14.79%), Poaceae (5.89%), and Pottiaceae (3.16%) (Figure 2A). Cyperaceae and Polygonaceae consistently constituted the major components for Kiang in both cold and warm seasons. At the genus level, the main plants foraged by Kiang in the warm season were *Carex* (48.43%), *Bistorta* (24.30%), *Poa* (5.62%), *Trigonotis* (5.36%), and *Sibbaldianthe* (3.55%); in the cold season, the main foraged plants include *Carex* (42.97%), *Bistorta* (17.57%), *Potentilla* (13.77%), *Poa* (5.03%), and *Didymodon* (2.98%) (Figure 2B). The dietary composition of kiang differed significantly between the warm and cold seasons (*χ* ^2^ = 481.342, df = 30, *p* < 0.001).

Similarly, for the Tibetan Gazelle, 27 orders, 40 families, and 58 genera of plants were identified in the warm‐season fecal samples and 24 orders, 35 families, and 56 genera of plants were identified in the cold‐season fecal samples. The primary taxa consumed by Tibetan Gazelle in the warm season include Polygonaceae (48.30%), Rosaceae (14.33%), Primulaceae (12.99%), Cyperaceae (6.00%), and Poaceae (3.57%); in the cold season, the main foraged plants were dominated by Ephedraceae (37.69%), Rosaceae (14.79%), Asteraceae (10.82%), Plantaginaceae (6.78%), and Caprifoliaceae (4.56%) (Figure 2C). At the genus level, the main forage plants by Tibetan Gazelle in the warm season include *Bistorta* (44.94%), *Lysimachia* (12.68%), *Sibbaldianthe* (11.84%), *Carex* (6.00%), and *Hedysarum* (3.24%); in the cold season, the main consumed plants include *Ephedra* (37.69%), *Potentilla* (14.10%), *Artemisia* (10.82%), *Lagotis* (4.79%), and *Phlomoides* (3.92%) (Figure 2D). The dietary composition of Tibetan Gazelle also differed significantly between the warm and cold seasons (*χ* ^2^ = 311.090, df = 25, *p* < 0.001).

Significant differences in plant species consumed by Kiang and Tibetan Gazelle were observed during both the warm (*χ* ^2^ = 157.641, df = 25, *p* < 0.001) and cold seasons (*χ* ^2^ = 182.280, df = 25, *p* < 0.001).

### 3.2 Comparison of Dietary Composition Diversity Between Kiang and Tibetan Gazelle

The α‐diversity analysis (Chao1, Shannon–Wiener diversity, Simpson's index, and Pielou's evenness) revealed that, in the warm season, a significant difference was detected between Kiang and Tibetan Gazelle in the Chao1 index, indicating greater dietary richness in one species. However, no significant differences were observed in the other three indices (Table 1). A similar pattern was observed in the cold season, with a significant interspecific difference in the Chao1 index but no significant differences in Shannon, Simpson, or Pielou indices (Table 1).

**TABLE 1:** α‐Diversity in the food composition of Kiang ( *Equus kiang* ) and Tibetan Gazelle ( *Procapra picticaudata* ) in the warm and cold seasons.

Index	Season	Kiang	Tibetan Gazelle	*p*
Chao1	Warm	17.99 ± 0.44	24.09 ± 0.69	≤ 0.001
	Cold	17.18 ± 0.71	14.98 ± 0.6	0.018
Shannon–Wiener	Warm	1.18 ± 0.05	1.32 ± 0.07	0.102
	Cold	1.46 ± 0.07	1.27 ± 0.08	0.081
Simpson	Warm	0.47 ± 0.02	0.45 ± 0.03	0.829
	Cold	0.39 ± 0.03	0.44 ± 0.03	0.240
Pielou	Warm	0.41 ± 0.02	0.42 ± 0.02	0.752
	Cold	0.52 ± 0.03	0.47 ± 0.03	0.210

**FIGURE 3:**
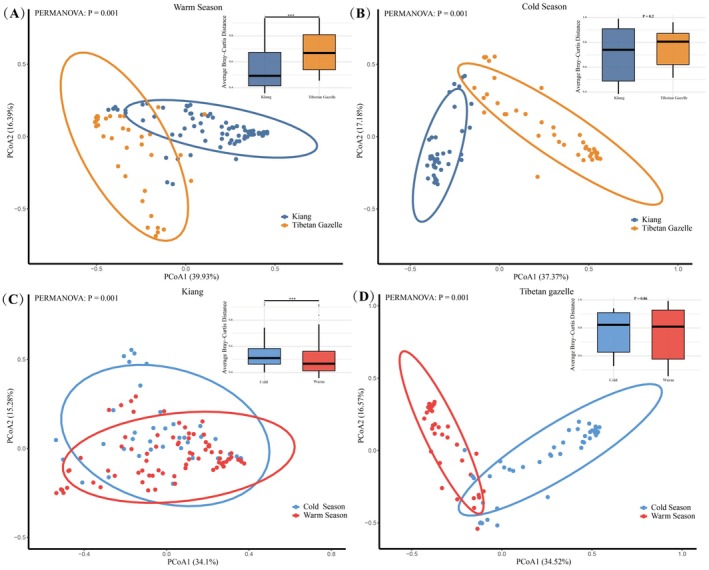
(A–D) Principal coordinates analysis (PCoA) based on Bray–Curtis dissimilarity illustrating differences in dietary composition between the Kiang ( *Equus kiang* ) and the Tibetan gazelle ( *Procapra picticaudata* ) across the warm and cold seasons, and the corresponding average inter‐individual dietary dissimilarity (Average Bray–Curtis Distance).

The principal coordinates analysis (PCoA) results showed that, during the warm season (Figure 3A), PERMANOVA results were highly significant (*p* = 0.001), indicating a clear multivariate differentiation in dietary composition between the Kiang and the Tibetan Gazelle. Consistently, the average Bray–Curtis distance differed markedly between species (*p* = 2.6 × 10^−9^), reflecting a pronounced overall dietary dissimilarity, with Tibetan Gazelles exhibiting higher inter‐individual variability in diet composition. In contrast, in the cold season (Figure 3B), PERMANOVA remained significant (*p* = 0.001) whereas the average Bray–Curtis distance did not differ significantly (*p* = 0.2), suggesting a directional shift in dietary composition between species but a comparable magnitude of overall dissimilarity.

In terms of within‐species seasonal variation, the Kiang (Figure 3C) exhibited significant differences in dietary composition between the cold and warm seasons (PERMANOVA *p* = 0.001), with the mean Bray–Curtis distance also showing a significant difference (*p* = 4.9 × 10^−5^), indicating a pronounced seasonal shift in diet and greater inter‐individual variation during the cold season. In contrast, the Tibetan gazelle (Figure 3D) showed a clear separation between seasons in the PCoA ordination (PERMANOVA *p* = 0.001), whereas the mean Bray–Curtis distance was not significant (*p* = 0.86), suggesting a directional shift in dietary composition across seasons, but with comparable levels of inter‐individual variation between the cold and warm seasons.

### 3.3 Difference Analysis of Food Composition

**FIGURE 4:**
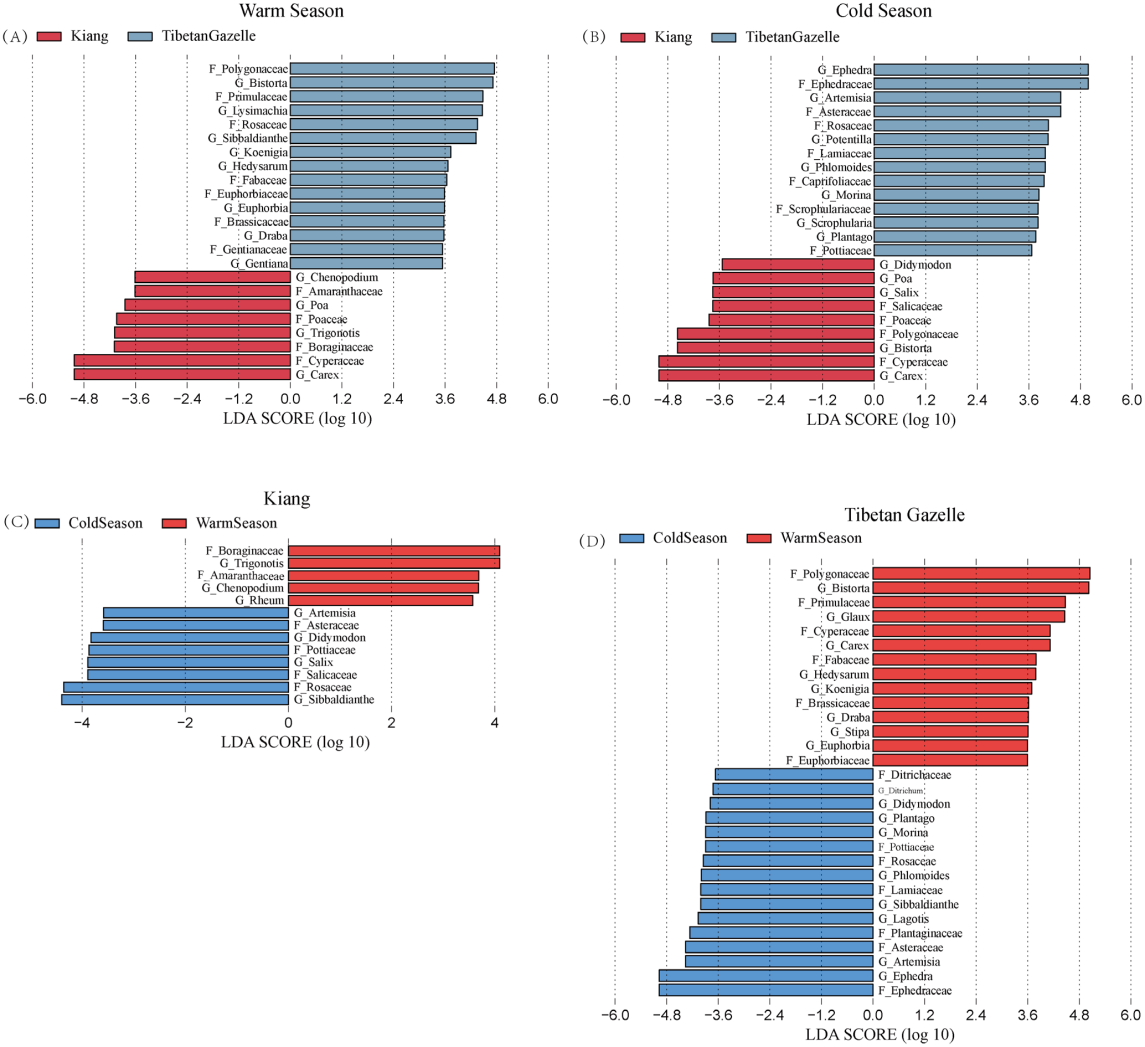
(A–D) Biomarkers of dietary composition identified by LEfSe analysis (LDA > 3.5, *p* < 0.05) for Kiang ( *Equus kiang* ) and Tibetan Gazelle ( *Procapra picticaudata* ).

LEfSe analysis (LDA > 3.5, *p* < 0.05) distinct dietary markers between Kiang and Tibetan Gazelle. During the warm season, Tibetan Gazelle exhibited significantly higher relative abundances of several plant taxa compared to Kiang, including, at the family level, Polygonaceae, Primulaceae, Rosaceae, Fabaceae, Euphorbiaceae, Brassicaceae, and Gentianaceae; and at the genus level, *Bistorta*, *Lysimachia*, *Sibbaldianthe*, *Koenigia*, *Hedysarum*, *Euphorbia*, *Draba*, and *Gentiana*. In contrast, Kiangs showed greater abundances of Amaranthaceae, Poaceae, Boraginaceae, and Cyperaceae at the family level, and *Chenopodium*, *Poa*, *Trigonotis*, and *Carex* at the genus level (Figure 4A). In the cold season, food taxa significantly enriched in Tibetan Gazelles included Ephedraceae, Asteraceae, Rosaceae, Lamiaceae, Caprifoliaceae, Scrophulariaceae, and Pottiaceae (family level), as well as *Ephedra*, *Artemisia*, *Potentilla*, *Phlomoides*, *Morina*, *Plantago*, and *Scrophularia* (genus level). Meanwhile, Kiang exhibited higher relative abundances of Salicaceae, Poaceae, Polygonaceae, and Cyperaceae at the family level, and *Didymodon*, *Poa*, *Salix*, *Bistorta*, and *Carex* at the genus level (Figure 4B).

The dietary composition of Kiang exhibited significant seasonal variation. At the family level, Boraginaceae and Amaranthaceae were significantly more abundant in the warm season, while Asteraceae, Pottiaceae, Salicaceae, and Rosaceae dominated in the cold season. At the genus level, *Trigonotis*, *Chenopodium*, and *Rheum* were more prevalent in the warm season, whereas *Artemisia*, *Didymodon*, *Salix*, and *Sibbaldianthe* showed higher abundances in the cold season (Figure 4C).

Similarly, Tibetan Gazelle also showed pronounced seasonal shifts in dietary composition. Families enriched in the warm season included Polygonaceae, Primulaceae, Cyperaceae, Fabaceae, Amaranthaceae, Brassicaceae, and Euphorbiaceae. Corresponding dominant genera were *Bistorta*, *Glaux*, *Carex*, *Hedysarum*, *Stipa*, *Koenigia*, *Draba*, *Euphorbia*, *Chenopodium*, and *Geum*. In contrast, during the cold season, Ditrichaceae, Pottiaceae, Rosaceae, Lamiaceae, Plantaginaceae, and Asteraceae became more prominent at the family level, while *Ditrichum*, *Didymodon*, *Morina*, *Plantago*, *Phlomoides*, *Sibbaldianthe*, *Lagotis*, and *Artemisia* were significantly enriched at the genus level (Figure 4D).

### 3.4 Dietary Niche Breadth and Overlap

At the genus level, dietary niche breadth (BA) declined for both species in the cold season. For Kiang, BA decreased from 0.49 in the warm season to 0.48 in the cold season, while for Tibetan Gazelle, it declined from 0.59 to 0.46. Tibetan Gazelle had a broader niche than Kiang in the warm season, whereas Kiang had a broader niche in the cold season (Table 2).

**TABLE 2:** Dietary niche breadth at the genus level for Kiang and Tibetan Gazelle.

Speacies	Season	Levins index (BA)
Kiang	Warm	0.49
	Cold	0.48
Tibetan Gazelle	Warm	0.59
	Cold	0.46

The dietary niche overlap index (*O*_*jk*_) between the two species was 0.89 in the warm season and slightly decreased to 0.87 in the cold season, indicating a consistently high degree of trophic overlap throughout the year (Table 3).

**TABLE 3:** Dietary niche overlap index at the genus level for Kiang and Tibetan Gazelle.

Species pair	Season	Pianka index (*O*_*jk*_)
Kiang and Tibetan Gazelle	Warm	0.89
Kiang and Tibetan Gazelle	Cold	0.87

## 4 Discussion

### 4.1 Dietary Differences and Seasonal Variations Between Kiang and Tibetan Gazelle

In this study, we used DNA metabarcoding to assess the dietary composition of sympatric Kiang and Tibetan Gazelle across seasons in the alpine ecosystem of Shiqu County, aiming to uncover mechanisms of coexistence along the nutritional niche dimension. Our results show that Cyperaceae and Polygonaceae dominated the diet of Kiangs in both warm and cold seasons, with Cyperaceae accounting for 48.43% and 42.97%, respectively. This pattern reflects a consistent foraging preference for graminoid plants with high fiber and low nutritional quality, a trait typical of large‐bodied hindgut fermenters like equids (Codron et al. 2007; Clauss et al. 2003). Similar dietary patterns have been reported in the Altun Mountains and Hoh Xil, where Kiangs predominantly consume Poaceae and Cyperaceae, particularly during resource‐constrained periods (Dong et al. 2015; Cao et al. 2009). The relatively high and stable proportions of Cyperaceae in both seasons suggest that Kiangs maintain a conservative foraging strategy focused on dominant graminoid species commonly found in alpine meadows (Qiu 2006), possibly due to their ability to efficiently digest low‐quality forage through gut microbial adaptations that enhance fiber degradation and energy acquisition (Liu et al. 2022).

In contrast, the Tibetan Gazelle exhibited more seasonal variation in diet composition. During the warm season, its diet was dominated by Polygonaceae (48.30%) and Rosaceae (14.33%), reflecting selective foraging on high‐quality herbaceous plants. In the cold season, however, Tibetan Gazelles shifted to more stress‐tolerant taxa such as Ephedraceae and Asteraceae, which are rich in secondary metabolites and better adapted to harsh environments (Zhang et al. 2024). This shift likely represents a nutritional trade‐off driven by seasonal decline in plant quality and availability, consistent with findings from Tianjun County and other alpine regions (Li and Jiang 2007). Such seasonal dietary shifts highlight the Tibetan Gazelle's flexible foraging strategy and its sensitivity to phenological dynamics of alpine vegetation. Although both species primarily consume herbaceous plants, differences in the identity and proportional use of key plant families and genera indicate dietary niche partitioning. This differentiation reduces direct resource competition and supports the long‐term coexistence of these sympatric herbivores under fluctuating environmental conditions.

Our α diversity analysis revealed significant seasonal shifts in the dietary composition of Kiangs and Tibetan Gazelles. In the cold season, Kiangs exhibited a significantly higher Chao1 index, indicating broader dietary diversity compared to Tibetan Gazelles. This suggests that Kiangs are more flexible in expanding their dietary niche to cope with resource scarcity during the harsh winter months. Conversely, Tibetan Gazelles demonstrated higher dietary diversity in the warm season, where plant availability is abundant, and their foraging preferences are less constrained. These seasonal shifts reflect the species' adaptive strategies to optimize resource use under varying ecological conditions (Kartzinel et al. 2015). Despite these differences, both species demonstrated significant overlap in dietary composition, particularly during the warm season, highlighting the shared reliance on common plant families such as Poaceae and Cyperaceae. Our β‐diversity results revealed that both species identity and season exerted significant effects on the dietary composition of Kiangs and Tibetan Gazelles. During the warm season, Tibetan Gazelles exhibited greater inter‐individual dietary dissimilarity than Kiangs, indicating higher foraging flexibility under conditions of abundant food resources. In contrast, Kiangs showed higher inter‐individual variation in diet composition during the cold season than in the warm season, suggesting that they cope with winter resource scarcity by increasing individual‐level dietary flexibility. This pattern is consistent with the findings of Harvey et al. (2024) in African large herbivores, which demonstrated that under fluctuating resource environments, dietary flexibility may represent a key mechanism facilitating species coexistence.

### 4.2 Dietary Niche Characteristics of Kiang and Tibetan Gazelle

Our results show that both species exhibited a decline in niche breadth (BA) during the cold season, with a more pronounced reduction in Tibetan Gazelles, indicating that their dietary diversity contracted under harsher winter conditions when plant availability was limited. This pattern is consistent with previous studies indicating reduced food resource heterogeneity in alpine rangelands during winter, leading to increased dietary specialization among herbivores (Caram et al. 2024; Song et al. 2020). Interestingly, Tibetan Gazelle exhibited a broader dietary niche than Kiang in the warm season (BA = 0.59 vs. 0.49), potentially due to its smaller body size and higher selectivity, which may allow it to exploit a wider array of herbaceous species during periods of greater plant availability (Demment 1985). In contrast, Kiang displayed a relatively broader niche in the cold season (BA = 0.48 vs. 0.46), which may be attributed to its generalist foraging behavior and greater mobility, allowing it to access scattered forage patches under snow‐covered or degraded winter conditions (Schaller 1998). Although Pianka's index indicated a high level of dietary niche overlap between Kiangs and Tibetan Gazelles in both seasons (0.89 in warm, 0.87 in cold), our results suggest that actual interspecific competition may be mitigated through niche partitioning. This pattern of “high overlap–low competition” can be explained by differentiation along the nutritional niche dimension, where each species exhibits distinct preferences for key plant taxa. LEfSe analysis demonstrated that Kiangs consistently consumed greater proportions of Cyperaceae, Poaceae, and Amaranthaceae, whereas Tibetan Gazelles relied more heavily on forbs such as Polygonaceae, Rosaceae, and Ephedraceae. These differences likely reflect each species' digestive physiology and foraging strategy: Kiangs, as large hindgut fermenters, efficiently process low‐quality but abundant graminoids (Clauss et al. 2003). Tibetan Gazelles, in contrast, as small‐bodied ruminants, prioritize nutrient‐rich and easily digestible dicotyledonous plants (Hofmann 1989). Moreover, spatial and behavioral segregation may further reduce interspecific competition (Li, Wang, et al. 2022). Tibetan Gazelles typically prefer montane shrublands with diverse forb communities, whereas Kiangs occupy open alpine meadows dominated by graminoids (Lu et al. 2005). Temporal differences in daily activity patterns—such as staggered foraging times—may also contribute to resource partitioning and facilitate coexistence under limited forage conditions (Li, Wang, et al. 2022).

In summary, our study reveals that both Kiangs and Tibetan Gazelles exhibit notable seasonal plasticity in their dietary composition and niche breadth; however, Tibetan Gazelles show stronger ecological adaptability than Kiangs. This flexibility enables them to adapt to the extreme seasonality and resource scarcity characteristic of alpine ecosystems on the eastern Qinghai–Tibet Plateau. Tibetan Gazelles exhibited a broader and more variable dietary niche than Kiangs in the warm season, indicating greater foraging plasticity under favorable conditions. In contrast, Kiangs maintained a more consistent diet dominated by graminoid species, reflecting a stable foraging strategy that likely supports their persistence in habitats with continuous graminoid availability. These findings highlight the ecological importance of niche differentiation. Such differentiation reduces direct interspecific competition and aligns with niche complementarity theory, which posits that resource partitioning facilitates species richness and community stability (MacArthur 1958; Griffin and Silliman 2011). Moreover, the observed individual‐level dietary variation in Kiangs and Tibetan Gazelles suggests that intraspecific niche variation may also play a role in facilitating coexistence and enhancing population resilience under fluctuating conditions (Bolnick et al. 2003).

However, ongoing climate change and human‐induced habitat alterations pose significant threats to these established foraging strategies (Malpeli et al. 2024). Changes in plant community composition, phenology, and productivity may shift food availability patterns, potentially disrupting current trophic relationships and intensifying interspecific competition (Jung et al. 2015; Schweiger et al. 2015). These dynamics are particularly concerning in ecologically sensitive areas such as the Three‐River‐Source region, where herbivore communities are already under pressure from rangeland degradation and climate variability (Gu et al. 2023; Wang, Wang, et al. 2025). Therefore, we recommend long‐term ecological monitoring of herbivore diets, habitat use, and vegetation dynamics as a foundation for adaptive management. Future research should adopt an integrative approach combining DNA metabarcoding, high‐resolution movement data, plant phenology monitoring, and habitat suitability modeling to explore how multiple niche dimensions shift under environmental change (Wang, Cao, et al. 2025). This will improve our ability to predict community assembly processes, inform conservation strategies, and support biodiversity persistence in fragile alpine grassland systems (Fluri et al. 2023).

### 4.3 Limitations and Future Directions

Although this study provides valuable insights into the seasonal dietary strategies and coexistence mechanisms of sympatric Kiang and Tibetan Gazelle, several limitations should be acknowledged. First, the sample collection was limited to two representative periods (the warm and cold seasons of 2023–2024), and this discrete temporal scale may not fully reflect dietary changes during transitional periods or under extreme climatic conditions such as severe winters or dry summers. Therefore, the results primarily represent seasonal characteristics rather than year‐round dynamics. Future studies should adopt continuous or multi‐year sampling frameworks to more accurately capture the temporal dynamics of food composition and trophic flexibility. Second, this study mainly focused on dietary composition; integrating behavioral observations, microhabitat use, and stable isotope analysis in future research would help to provide a more comprehensive understanding of how these two ungulate species partition resources across multiple ecological niche dimensions.

## Author Contributions

**Huiqin Dong:** data curation (equal), formal analysis (equal), writing – original draft (equal). **Bin Feng:** data curation (equal), writing – review and editing (equal). **Shuai Yang:** data curation (equal). **Yuna Jia:** writing – review and editing (equal). **Mingsen Qin:** resources (equal), supervision (equal), writing – review and editing (equal). **Wenke Bai:** resources (equal), supervision (equal), writing – review and editing (equal).

## Conflicts of Interest

The authors declare no conflicts of interest.

## Data Availability

The raw sequence reads have been deposited in the NCBI Sequence Read Archive (SRA) under BioProject accession PRJNA1364803. Data supporting the dietary analyses have been deposited in the Dryad Digital Repository at https://doi.org/10.5061/dryad.g79cnp63c.
